# Evaluation of pre-operative endometrial biopsy accuracy and etiological distribution in abnormal uterine bleeding using the PALM–COEIN classification

**DOI:** 10.3389/fmed.2026.1784420

**Published:** 2026-02-24

**Authors:** Rulin Deniz, Yakup Baykus, Alihan Tigli, Muhammet Bora Uzuner, Nazli Sener, Yasemin Ercan Degirmenci, Guzide Ece Akinci, Erdem Gurkan, Oguzhan Karakoc, Mustafa Ata Aydin, Elif Emre, Suleyman Aydin

**Affiliations:** 1 Department of Obstetrics and Gynecology, Faculty of Medicine, Bandirma Onyedi Eylül University, Balikesir, Türkiye; 2 Department of Anatomy, Faculty of Medicine, Bandirma Onyedi Eylül University, Balikesir, Türkiye; 3 Gynecology and Obstetrics Clinic, Bandirma Training and Research Hospital, Bandirma, Balikesir, Türkiye; 4 School of Medicine, Clinical Sciences, Gazi University, Ankara, Türkiye; 5 Department of Medical Anatomy, Faculty of Medicine, Firat University, Elazig, Türkiye; 6 Department of Medical Biochemistry, Faculty of Medicine, Firat University, Elazig, Türkiye

**Keywords:** abnormal uterine bleeding, diagnostic accuracy, endometrial biopsy, hysterectomy, PALM-COEIN

## Abstract

**Objective:**

Abnormal uterine bleeding (AUB) is one of the leading causes of gynecological consultations and indications for hysterectomy. The aim of this study is to determine the distribution of aetiological causes in patients undergoing hysterectomy due to AUB according to the FIGO PALM-COEIN system and to evaluate the diagnostic concordance between pre-operative endometrial biopsy (EB) results and final hysterectomy pathology.

**Methods:**

This retrospective, descriptive and analytical study included 296 patients who underwent hysterectomy due to AUB at Bandirma Onyedi Eylül University Hospital between 2020 and 2025. The patients' demographic data, clinical characteristics, and histopathological results were examined from hospital records. The causes of AUB were categorized according to the PALM-COEIN system (Polyps, Adenomyosis, Leiomyoma, Malignancy; Coagulopathy, Ovulatory, Endometrial, Iatrogenic, Unclassifiable). The concordance of pre-operative EB results with final pathology was analyzed using sensitivity, specificity, positive/negative predictive values, the Kappa coefficient, and ROC analysis AUC.

**Results:**

The mean age of the patients was 48.2 ± 6.9 years. According to the PALM-COEIN classification, structural causes (PALM) were the most common etiology, with leiomyomas (35.5% isolated, 59.8% total) forming the pre-dominant group. The rate of post-menopausal bleeding was determined to be 28.0%. Regarding concordance between pre-operative EB and final pathology, EB demonstrated high specificity but limited sensitivity, particularly for focal lesions. For AEH+ (atypical endometrial hyperplasia and above), sensitivity was 22.2%, specificity was 99.6%, and AUC was 0.609; for endometrial polyps, sensitivity was 30.6%, specificity was 83.0%, and AUC was 0.568. Overall agreement between biopsy and final pathology (Kappa) was low to moderate, and ROC analysis indicated limited discriminatory ability across subgroups.

**Conclusion:**

Among patients undergoing hysterectomy for AUB, structural pathologies, particularly leiomyomas, constitute the primary reason for hysterectomy. Although pre-operative EB is successful in ruling out pathologies, it may be insufficient in detecting focal lesions and some pre-malignant conditions due to its low sensitivity. Therefore, EB results should be supported by advanced diagnostic imaging methods, especially when clinical suspicion exists during the decision-making process for hysterectomy.

## 1 Introduction

Abnormal uterine bleeding (AUB) is a clinical condition characterized by uterine bleeding originating from the uterine corpus in the absence of pregnancy, which is defined by an abnormal menstrual pattern in terms of regularity, frequency, duration, or amount of bleeding (1, 2). AUB is one of the most common reasons for gynaecological consultations in women and poses a significant physical, psychosocial and economic burden both during the reproductive years and in the perimenopausal and post-menopausal periods (1, 3, 4). Furthermore, AUB remains one of the most common indications for hysterectomy worldwide (3, 5).

The heterogeneous nature of AUB aetiology and the inconsistency of definitions used in the past have complicated the comparability of clinical studies and patient management (6). To address this issue, the International Federation of Gynaecology and Obstetrics (FIGO) has developed the universally accepted PALM-COEIN classification system for AUB. This system categorises the causes of AUB into two main groups: structural (PALM) and non-structural (COEIN). Structural causes are defined as polyps (AUB-P), adenomyosis (AUB-A), leiomyoma (AUB-L), and malignancy/hyperplasia (AUB-M), while non-structural causes include coagulopathy (AUB-C), ovulatory dysfunction (AUB-O), endometrial causes (AUB-E), iatrogenic causes (AUB-I), and causes that have not yet been classified (AUB-N) (7–9). The PALM-COEIN system enables the identification of multiple aetiologies in the same patient through both clinical evaluation and imaging and histopathological examinations.

Post-menopausal bleeding (PMB) is one of the clinical presentations where the risk of endometrial carcinoma is significant, unlike bleeding during the reproductive years. Although the FIGO PALM-COEIN system was designed for the reproductive period, the accuracy of pre-operative diagnosis in PMB cases, which account for a significant proportion of hysterectomy indications, is critical in determining the limits of surgical planning. Therefore, in studies evaluating the diagnostic power of endometrial biopsy (EB) in the management of AUB, it is important to specifically examine this high-risk group.

EB is widely used in the investigation of AUB aetiology due to its minimally invasive, easily applicable and cost-effective nature (10). However, the extent to which biopsy results reflect the final hysterectomy pathology remains controversial due to factors such as the endometrium potentially containing focal lesions, inadequate sampling, and differences in histopathological interpretation (11). Studies evaluating the concordance between pre-operative EB and histopathological results of hysterectomy specimens have revealed that diagnostic discrepancies are significant, particularly in cases of hyperplasia and early-stage endometrial cancer (12). Studies evaluated within the PALM-COEIN framework in the literature also show that the sensitivity of pre-operative endometrial sampling is generally moderate. It has been reported that diagnostic upgrading or downgrading is frequently observed, particularly in cases of hyperplasia and early-stage endometrial cancer (10). In this context, comparing pre-operative EB results with the final hysterectomy pathology is important for evaluating diagnostic accuracy and optimising clinical decision-making processes. In particular, this correlation must be analysed in detail to prevent unnecessary hysterectomies, adopt more conservative treatment approaches in suitable patients, and accurately predict the risk of malignancy.

Hysterectomy provides a definitive solution in the treatment of AUB, but it is a major surgical procedure and carries significant perioperative and long-term complication risks (13, 14). Therefore, ensuring accurate diagnostic classification in patients prior to the decision for hysterectomy is of great importance, both to prevent unnecessary surgery and to avoid overlooking the risk of malignancy. Determining the extent to which pre-operative EB results correlate with the final hysterectomy pathology will contribute to rationalising clinical decision-making processes (15).

The aim of this study is to evaluate the distribution of indications within the PALM-COEIN classification system in patients who have undergone hysterectomy due to AUB, and to assess the concordance between pre-operative EB results and final hysterectomy pathology findings in post-menopausal and reproductive-age patients. Within this scope, it is intended to contribute to the literature regarding the clinical importance of pre-operative histopathological evaluation in the management of AUB by demonstrating the diagnostic performance of EB.

## 2 Materials and methods

### 2.1 Study design and participants

**Figure 1:**
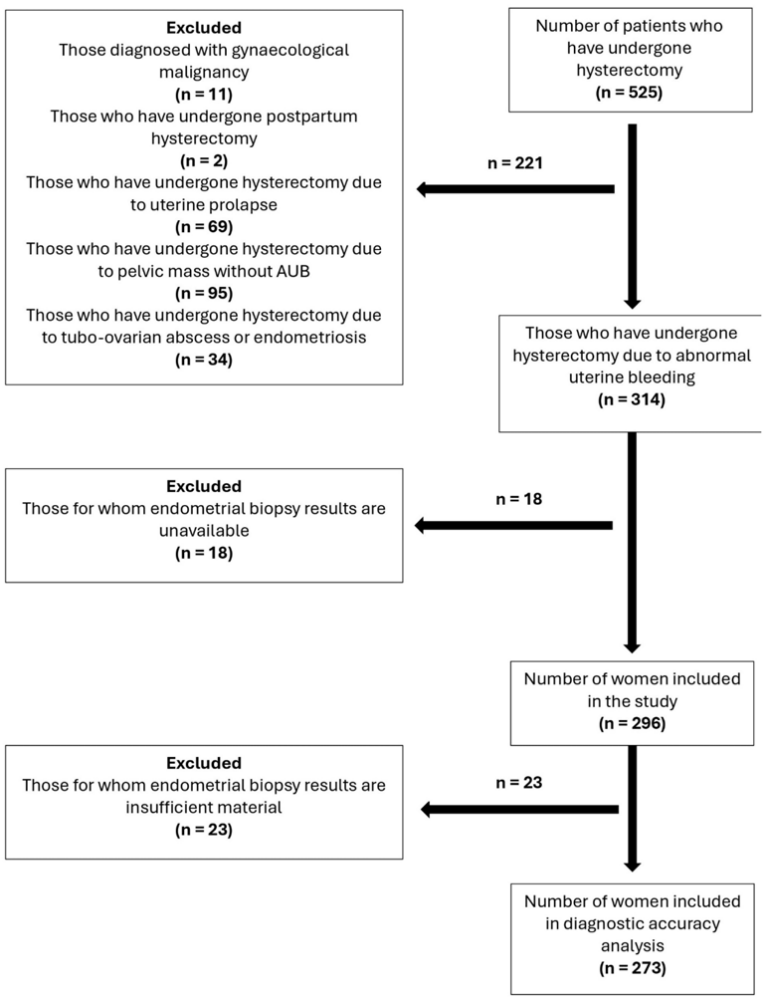
Flow chart for patients undergoing hysterectomy. The diagram illustrates the exclusion of cases with insufficient biopsy material (*n* = 23) from the final diagnostic accuracy analysis set (*n* = 273).

This study was designed as a retrospective, descriptive, and analytical research. The clinical records of patients who underwent hysterectomy due to AUB at Bandirma Onyedi Eylül University Hospital between 2020 and 2025 and who had not received a pre-operative diagnosis of gynaecological malignancy were examined. Clinical information and pathology reports (EB and hysterectomy) were retrospectively evaluated. After applying inclusion and exclusion criteria, a total of 296 patients were included in the study (Figure 1).

### 2.2 Endometrial sampling method

Pre-operative endometrial sampling was performed based on a standardized clinical protocol. The Pipelle cannula was used as the primary first-line method in the office setting for patients with suitable cervical patency and tolerance. Fractionated dilatation and curettage (D&C) under sedation was reserved for cases where office biopsy failed, cervical stenosis prevented catheter insertion, or the initial office sampling yielded insufficient tissue for diagnosis. All procedures were performed blindly without hysteroscopy guidance. The samples obtained were fixed in 10% neutral formalin and sent to the pathology laboratory, where they were reported according to World Health Organisation (WHO) criteria. Regarding data handling, cases with ‘insufficient material' (*n* = 23) were excluded from diagnostic accuracy calculations (sensitivity and specificity) to ensure statistical reliability.

### 2.3 Data collection tools

The study data were collected using a patient information form prepared in Excel format. This form recorded age, EB result, indication for hysterectomy, final hysterectomy pathology result, treatments administered for AUB, gynaecological examination and radiology findings, and surgical method information obtained from the hospital information management system. Patient identities were kept confidential and data were recorded using anonymous patient codes.

### 2.4 Statistical analysis

The data were analysed using the SPSS 23.0 statistical package. The distribution of demographic and clinical characteristics of the study group, the distribution of AUB and PMB according to the PALM–COEIN classification, and the distribution of histopathological findings in hysterectomy specimens according to anatomical region were summarised in terms of numbers and percentages and presented in graphs. In the evaluation of the diagnostic performance of pre-operative endometrial biopsy, hysterectomy pathology was accepted as the gold standard, and sensitivity, specificity, positive predictive value (PPV), negative predictive value (NPV), and accuracy (ACC) were calculated. ROC curves were constructed to evaluate the diagnostic discriminatory power of pre-operative endometrial biopsy, the area under the curve (AUC) was calculated for each pathology category, and AUC values were interpreted as moderate (0.70–0.80), good (0.80–0.90), and excellent (≥0.90) diagnostic accuracy (16). The agreement between pre-operative endometrial biopsy and hysterectomy pathology results was assessed using Cohen's kappa coefficient. Kappa values were categorised to describe the level of agreement as follows: 0–0.10, virtually no agreement; 0.11–0.40, slight agreement; 0.41–0.60, fair agreement; 0.61–0.80, moderate agreement; and 0.81–1.00, substantial agreement (17). A significance level of *P* < 0.05 was accepted for statistical tests.

### 2.5 Ethical approval

The study was conducted in accordance with the ethical principles outlined in the Helsinki Declaration. Ethical approval for the study was obtained from Bandirma Onyedi Eylul University Health Sciences Non-Interventional Research Ethics Committee (Date: 01.12.2025, Decision No: 2025-209). Due to the retrospective design of the study, the requirement for written informed consent was waived by the Ethics Committee in accordance with national legislation and institutional requirements.

## 3 Results

A total of 296 patients were included in the study. The demographic and clinical characteristics, including age distribution, clinical presentation, surgical procedures, and pre-operative medical management, are summarized in Table 1.

**Table 1:** Demographic and clinical characteristics of the study population (*n* = 296).

**Variable**	**Category**	***n***	**%**
Age distribution (years)	< 40	19	6.4
	41–50	207	69.9
	51–60	46	15.5
	61–70	21	7.1
	>70	3	1.0
Type of surgery	TAH	192	64.9
	TLH	102	34.5
	VH	2	0.7
Pre-operative treatment	COC	14	4.7
	D&C	4	1.4
	LNG-IUS	13	4.4
	NSAID	9	3.0
	Oral progesterone	60	20.3
	Not treated	196	66.2

TLH, total laparoscopic hysterectomy; TAH, total abdominal hysterectomy; VH, vaginal hysterectomy; COC, combined oral contraceptive; D&C, dilation and curettage; LNG-IUS: levonorgestrel-releasing intrauterine system; NSAID, non-steroidal anti-inflammatory drug.

The mean age of the patients was 48.2 ± 6.9 years, with the majority (69.9%) falling into the 41–50 age group. Regarding surgical procedures, Total Abdominal Hysterectomy (TAH) was the most frequently performed operation (64.9%), followed by Total Laparoscopic Hysterectomy (TLH; 34.5%). In terms of pre-operative management, the majority of patients (66.2%) proceeded directly to surgery without receiving prior medical treatment.

According to the PALM–COEIN classification, structural causes (PALM) constituted the majority of AUB cases. Among these, leiomyoma (AUB-L) was the most frequent etiology, accounting for 35.5% (*n* = 105) of patients. Combined structural categories were also observed, including AUB-L, A in 12.5% (*n* = 37) and AUB-L, *P* in 7.1% (*n* = 21) of cases. Other structural causes, such as adenomyosis (AUB-A) and malignancy and hyperplasia (AUB-M), were less common, each representing less than 5% of the study population.

Regarding non-structural causes (COEIN), the overall frequency was low. Ovulatory dysfunction (AUB-O) was identified in 1.0% (*n* = 3) of patients, while endometrial (AUB-E) and iatrogenic (AUB-I) causes were each observed in 0.3% (*n* = 1) of cases. Other non-structural or mixed categories accounted individually for less than 3% of the total cohort.

Within the PALM–COEIN classification framework, leiomyoma was the most frequently identified structural abnormality, being present in 59.8% (*n* = 177) of cases when both isolated and combined categories were considered. Adenomyosis and polyp were also commonly observed, occurring in 18.6% (*n* = 55) and 12.5% (*n* = 37) of patients, respectively. These findings reflect the cumulative burden of PALM-related structural causes in the study population. PMB cases were evaluated as a distinct clinical entity and were therefore excluded from the PALM–COEIN–based analysis.

Our study included 83 patients (28.0%) who presented with PMB. As PMB is not included in the FIGO PALM-COEIN classification system, it was evaluated as a separate aetiological group in the study. None of the 83 patients in this group, who had pre-operative endometrial biopsies reported as benign or insufficient material, had endometrial carcinoma or malignancy detected in their final hysterectomy pathology.

**Figure 2:**
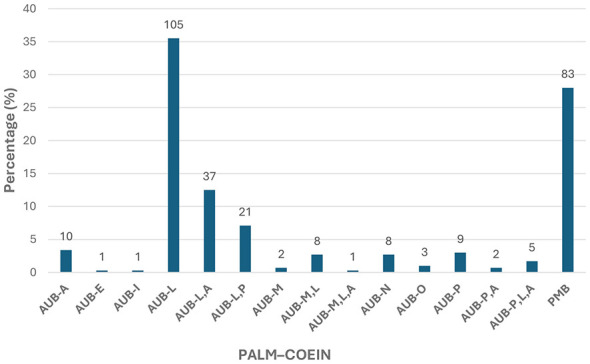
Distribution of AUB according to the PALM–COEIN classification and PMB. AUB, abnormal uterine bleeding; PALM–COEIN, polyp, adenomyosis, leiomyoma, malignancy and hyperplasia–coagulopathy, ovulatory dysfunction, endometrial, iatrogenic, not otherwise classified; PMB, post-menopausal bleeding.

The distribution of AUB etiologies according to the PALM–COEIN classification is illustrated in Figure 2.

Histopathological findings of hysterectomy specimens were evaluated for the uterus, cervix, ovaries, and fallopian tubes. Regarding uterine and myometrial pathology, the most frequent finding was leiomyoma, observed in 223 patients (75.3%). Adenomyosis was detected in 77 patients (26.0%). Normal histopathological findings were reported in 73 patients (24.7%) for the uterus and in 219 patients (74.0%) for the myometrium. In addition to uterine pathologies, incidental findings in the cervix and adnexa were recorded. Chronic cervicitis was the most common cervical finding (71.3%). High-grade cervical intraepithelial lesions (CIN II–III) were detected in 2.4% (*n* = 7) and invasive cervical carcinoma in 0.3% (*n* = 1) of patients. Regarding adnexal pathologies, serous cystadenoma (77.7%) and paratubal cysts (32.8%) were frequently observed. Occult ovarian malignancy/borderline tumors were rare (0.6%). Given that the primary focus of this study is endometrial pathology, detailed distributions of these incidental findings have been summarized here rather than presented in extensive tables.

**Figure 3:**
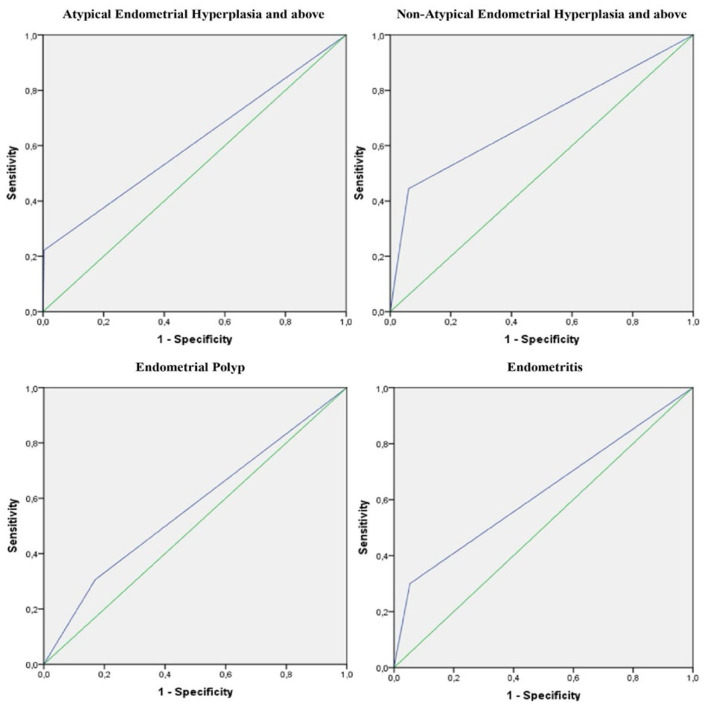
ROC curves of pre-operative endometrial biopsy findings for predicting final hysterectomy pathology.

The diagnostic performance of the pre-operative endometrial biopsy evaluated by ROC curve analysis for final hysterectomy pathology prediction is shown in Table 2 and Figure 3. In the group of Atypical Endometrial Hyperplasia and above (AEH+), the sensitivity of pre-operative biopsy was 22.2% and specificity was 99.6%, indicating that while the biopsy missed a substantial proportion of positive cases, it accurately identified the majority of normal cases. The PPV was 66.7%, the NPV was 97.4%, ACC was 97.1%, Cohen's Kappa coefficient was 0.322 and an AUC was 0.609, reflecting low to moderate agreement between pre-operative and final pathology. In the Non-Atypical Endometrial Hyperplasia and above (Non-AEH+) group, sensitivity was 44.4% and specificity was 93.9%, with a PPV of 20.0%, a NPV of 98.0%, an ACC of 92.3, a Kappa of 0.241 and an AUC of 0.692, indicating limited diagnostic accuracy for detecting positive cases while maintaining high accuracy for normal findings. For endometrial polyps, pre-operative biopsy demonstrated a sensitivity of 30.6% and a specificity of 83.0%, with a PPV of 28.3%, a NPV of 84.5%, an ACC of 73.6%, a Kappa of 0.132 and an AUC of 0.568 suggesting low sensitivity and modest concordance with final pathology. In the endometritis group, sensitivity was 30.0% and specificity was 94.7%, with a PPV of 17.6%, a NPV of 97.3%, an ACC of 92.3%, a Kappa of 0.185 and an AUC of 0.623, showing that pre-operative biopsy correctly identified most normal cases but detected only a limited proportion of endometritis cases. Overall, these results indicate that pre-operative EB demonstrates high specificity across different pathologies but variable sensitivity, and agreement with final hysterectomy pathology ranges from low to moderate depending on the lesion type.

**Table 2:** Concordance and diagnostic performance of pre-operative endometrial biopsy with final hysterectomy pathology (*n* = 273)^**^.

			**Endometrial pathology result of hysterectomy** ^*^		**Se (%)**	**Sp (%)**	**PPV (%)**	**NPV (%)**	**AUC (%)**	**ACC (%)**	**Kappa (SE)**
			**Normal (*n*)**	**AEH + (*n*)**							
Pathology result of endometrial biopsy	AEH +	Normal	263	7	22.2	**99.6**	**66.7**	97.4	0.609 (0.393–0.825)	**97.1**	0.322 (0.175)
		AEH +	1	2							
		Normal	AEH +								
	Non-AEH +	Normal	248	5	**44.4**	93.9	20.0	**98.0**	**0.692** (0.481–0.903)	92.3	0.241 0.110
		Non-AEH +	16	4							
		Normal	Endometrial polyp								
	Endometrial polyp	Normal	186	34	30.6	83.0	28.3	84.5	0.568 (0.476–0.661)	73.6	0.132
		Endometrial polyp	38	15							0.068
		Normal	Endometritis								
	Endometritis	Normal	249	7	30.0	94.7	17.6	97.3	0.623 (0.421–0.826)	92.3	0.185
		Endometritis	14	3							0.109

AEH+, atypical endometrial hyperplasia and above; NAEH+, non-atypical endometrial hyperplasia and above; Se (%): sensitivity (%); Sp (%), specificity (%); PPV (%), positive predictive value (%); NPV (%), negative predictive value (%); AUC, area under the curve; CI, confidence interval; Kappa, cohen's kappa coefficient; SE, standart error, ACC, accuracy. Bold values indicate the highest values.

^*^Final hysterectomy pathology was considered as the reference standard.

^**^Patients with insufficient material obtained from endometrial biopsy were excluded from the statistics.

The concordance between pre-operative EB findings and final hysterectomy histopathology is presented in Table 3. Among patients diagnosed with atypical hyperplasia on pre-operative biopsy (*n* = 3), the final histopathological diagnoses were adenocarcinoma, atypical hyperplasia, and non-atypical hyperplasia, each observed in one patient (33.3%).

**Table 3:** Comparison of pre-operative endometrial biopsy and final endometrial histopathology in hysterectomy specimens.

	**Endometrial pathology result of hysterectomy**							
**Pre-operative endometrial biopsy**	**Endometrial carcinoma**	**AEH**	**Non-AEH**	**Atrophic endometrium**	**Functional endometrium**	**Endometrial Polyp**	**Endometritis**	**Total**
AEH	1 (33.3%)	1 (33.3%)	1 (33.3%)	0	0	0	0	3
Non-AEH	0	2 (11.8%)	2 (11.8%)	0	10 (58.8%)	3 (17.6%)	0	17
Atrophic endometrium	0	0	0	0	0	1 (100%)	0	1
Functional endometrium	1 (0.5%)	2 (1.1%)	8 (4.4%)	6 (3.3%)	132 (72.5%)	28 (15.4%)	5 (2.7%)	182
Endometrial polyp	1 (1.9%)	1 (1.9%)	1 (1.9%)	4 (7.5%)	29 (54.7%)	15 (28.3%)	2 (3.8%)	53
Endometritis	0	0	1 (5.9%)	1 (5.9%)	10 (58.8%)	2 (11.8%)	3 (17.6%)	17
Insufficient sample	0	0	1 (4.3%)	7 (30.4%)	9 (39.1%)	5 (21.7%)	1 (4.3%)	23
Total (*n*)	3	6	14	18	190	54	11	296

AEH, atypical endometrial hyperplasia and above; Non-AEH, non-atypical endometrial hyperplasia and above.

In cases with a pre-operative diagnosis of non-atypical hyperplasia (*n* = 17), the most frequent final diagnosis was functional endometrium in 10 patients (58.8%), followed by endometrial polyp in three patients (17.6%), while atypical hyperplasia and non-atypical hyperplasia were each confirmed in two patients (11.8%).

Only one patient was diagnosed with atrophic endometrium on biopsy, which corresponded to an endometrial polyp on final pathology.

Among patients with a pre-operative diagnosis of functional endometrium (*n* = 182), the final hysterectomy specimens most commonly revealed functional endometrium in 132 patients (72.5%). However, endometrial polyps were identified in 28 patients (15.4%), non-atypical hyperplasia in eight patients (4.4%), atrophic endometrium in six patients (3.3%), and endometritis in five patients (2.7%). Notably, adenocarcinoma was detected in one patient (0.5%) within this group.

In patients with a pre-operative diagnosis of endometrial polyp (*n* = 53), the final histopathology confirmed endometrial polyp in 15 patients (28.3%), whereas functional endometrium was identified in 29 patients (54.7%).

Among cases diagnosed with endometritis on biopsy (*n* = 17), the most frequent final diagnosis was functional endometrium in 10 patients (58.8%), followed by endometritis in three patients (17.6%).

In the pre-operative evaluation, the biopsy result was reported as ‘insufficient material' in 23 patients (7.8%). When the final hysterectomy specimens of these cases were examined, functional endometrium (*n* = 9; 39.1%) and atrophic endometrium (*n* = 7; 30.4%) were detected in the vast majority, and no malignancy or atypical hyperplasia was found in this group. The relatively high rate of atrophic endometrium in this group suggests that the technical difficulty in sampling may be due to tissue scarcity. As the ‘insufficient material' result in the sample reflects sampling limitations rather than a specific histopathological diagnosis, these cases were excluded from the calculations to prevent statistical bias in diagnostic accuracy (sensitivity and specificity) analyses.

## 4 Discussion

AUB is a common condition that causes heterogeneous clinical symptoms due to different aetiological causes. Therefore, it requires a comprehensive diagnosis and treatment strategy (18, 19). The FIGO PALM-COEIN classification system provides a standard framework for categorising these reasons (7, 15). This study retrospectively analysed patients who underwent hysterectomy due to AUB, examining demographic factors, history of treatment failure, surgical methods, and accompanying gynaecological pathologies. This comprehensive clinical and pathological assessment will provide an important basis for understanding the diagnostic accuracy and performance of pre-operative EB against hysterectomy histopathology (20).

### 4.1 Demographic findings

The demographic structure of our study largely reflects the population undergoing hysterectomy due to AUB. Consistent with the literature, 69.9% of patients (*n* = 207) were in the perimenopausal period and concentrated in the 41–50 age range (21). AUB in this age group is generally associated with hormonal fluctuations and prolonged exposure to oestrogen. Therefore, particular attention should be paid to endometrial hyperplasia and endometrial cancer in this age group (22). The low prevalence among women under 40 years of age (6.4%) indicates the clinical priority of fertility-preserving treatment in young patients (5).

Post-menopausal patients have been classified as a separate category from the PALM-COEIN classification due to their different aetiological spectrum, high likelihood of endometrial carcinoma, and being outside the reproductive age. Over 90% of malignancies seen in this patient group present with AUB (23).

### 4.2 Surgical method preferences

In our study, the most frequently preferred surgical method was total abdominal hysterectomy (TAH) at 64.9%. This was followed by total laparoscopic hysterectomy (TLH) at 34.5% and vaginal hysterectomy (VH) at 0.7%.

Although the literature emphasises that the vaginal approach (VA) should be preferred for benign hysterectomies due to its minimally invasive nature, cost-effectiveness and rapid recovery process (24, 25), in our study, the VH ratio was limited to 0.7%. One of the main reasons for this low rate is that patients who underwent surgery solely for uterine prolapse were excluded from our study design. Furthermore, the detection of leiomyoma in 75.3% of the final pathology reports in our series suggests that the size and volume of the uterus in this patient group may have made vaginal extraction technically challenging. Being a tertiary centre, the frequency of referred complex cases with a high risk of adhesions and large uterine volumes has shifted the surgical preference towards abdominal or laparoscopic approaches rather than the vaginal route.

### 4.3 Pre-operative medical treatment

Our study found that 66.2% of patients underwent surgery directly without receiving any medical treatment for AUB. This suggests that urgent hysterectomy may be necessary due to severe symptoms, structural pathologies, or contraindications to medical treatment (26). The most commonly administered treatments among patients receiving medical treatment were oral progesterone (20.3%), combined oral contraceptives (4.7%) and levonorgestrel-releasing intrauterine system (LNG-IUS; 4.4%). It is known that these treatment methods eliminate the need for hysterectomy in some women (27–29). However, the fact that hysterectomy is ultimately required despite these treatments indicates that medical treatment has limited long-term efficacy in a significant proportion of women with AUB, particularly those with underlying structural pathology (30, 31).

### 4.4 Etiological distribution according to the PALM-COEIN classification

According to the FIGO PALM-COEIN classification system, surgery is significantly more frequently performed in cases with structural aetiologies (PALM). Hysterectomy remains an important option in the treatment of cases with identifiable structural abnormalities (3, 32).

In our study, leiomyoma (AUB-L) was the most common indication for hysterectomy, accounting for 35.5%. Leiomyoma could be isolated or observed in conjunction with both adenomyosis (AUB-L, A) and polyps (AUB-P, L). When all categories containing leiomyoma are included, this rate reaches 60%. Similar results have been reported in many studies in the literature (33–35). These findings emphasise the importance of leiomyoma aetiology in the pathogenesis of severe AUB requiring surgical intervention (31). In our study, AUB-A (3.4%) and AUB-P (3%) were observed as other common indications for isolated hysterectomy after AUB-L. Although prevalence rates vary in studies conducted in different regions, adenomyosis and endometrial polyps play a significant role in the aetiology of hysterectomy (35, 36).

Non-structural causes (COEIN) generally account for a small proportion of hysterectomy cases associated with AUB (8). In our study, the rate of hysterectomies performed for non-structural reasons was found to be only 4.4% (*n* = 13). The clear pre-dominance of structural aetiologies supports the view that hysterectomy should be used in the treatment of patients with complex and identifiable structural causes of AUB, rather than purely for functional disorders (37, 38).

### 4.5 Histopathological findings of hysterectomy specimens

The histopathological examination of hysterectomy specimens in the studies conducted also confirms the pre-dominance of structural lesions (39, 40). In our study, leiomyoma was detected in 75.3% of hysterectomy pathologies and adenomyosis in 26%. In the literature, the prevalence of leiomyoma ranges from 22 to 80%, depending on age, hysterectomy indication, and ethnic origin (41–43), The prevalence of adenomyosis is reported to be between 8 and 61.5% (44, 45). These two pathologies constitute the majority of uterine findings in hysterectomy specimens.

When evaluating the final pathology results in our study, functional or benign histological patterns were observed in a significant portion of the endometrium (82.3%; *n* =273), while chronic cervicitis was the most common cervical diagnosis (71.3%; *n* = 211). Incidental cervical lesions detected in the final histopathological examination demonstrate that the cervix should never be neglected during the pre-operative evaluation process. The detection of high-grade intraepithelial lesions (CIN II-III) in 2.4% (*n* = 7) and invasive cancer in 0.3% (*n* = 2) of the cervix in our study demonstrates that the risk of occult malignancy or pre-malignancy cannot be disregarded even in AUB cases operated on for benign indications. These findings are consistent with the rates of occult cervical cancer reported in the large-scale studies by Desai et al (46). In light of the data we have obtained, pre-operative cervical smear screening should be considered not merely as a ‘recommended' test, but as a mandatory step in AUB management that can directly influence the scope of the surgical procedure (whether the cervix will be preserved or not).

Similarly, in the histopathological evaluation of ovaries, non-benign pathologies were incidentally detected in 0.6% of cases (*n* = 2). Desai et al. reported the rate of occult ovarian cancer as 0.19% (46). Therefore, it is critically important to perform advanced pre-operative investigations for ovarian pathologies in suspected patients (47). Our study found benign pathologies in all salpiks, and similar results have been reported in the literature (48–50). Our data indicate that AUB requiring hysterectomy is generally caused by multifactorial reasons involving uterine, myometrial, cervical and adnexal pathology rather than isolated endometrial disease.

### 4.6 Diagnostic performance of endometrial biopsy

The comparison between pre-operative EB findings and hysterectomy pathology is of critical importance for evaluating diagnostic accuracy and constitutes one of the primary focal points of clinical research. The accuracy of EB diagnosis largely depends on the pathological threshold value used to define the disease and demonstrates a classic balance between sensitivity and specificity (51).

When the diagnostic threshold is broadened to include atypical hyperplasia, a moderate increase in sensitivity is generally observed, allowing for the detection of a wider range of endometrial changes. However, this trade-off inherently leads to a decrease in specificity and often results in a low positive predictive value (PPV), thereby increasing the false-positive rate (52).

Our study generally yielded results consistent with the literature. When the threshold value for pre-operative EB was set at AEH+, specificity reached 99.6%, while sensitivity was found to be 22.2%. When the threshold value was set at Non-AEH and above, sensitivity increased to 44.4%, while specificity decreased to 93.9%.

The low sensitivity rate of 22.2% observed for AEH and above lesions identified in our study indicates that the risk of ‘false negatives' in pre-operative EB is a factor that must be considered in clinical practice. The fact that the pre-operative biopsy results were reported as benign in two of the three cases in which endometrial carcinoma was detected in the final pathology supports the limitation of blind sampling methods, particularly in detecting focal malignant or pre-malignant lesions.

The diagnostic performance of pre-operative endometrial biopsy (EB) subgroups in predicting final hysterectomy endometrial pathology was assessed using ROC analysis (**Table 2, Figure 3**). Overall, the discriminatory ability of EB was limited across all subgroups, with AUC values below 0.700. In particular, for endometrial polyps and endometritis, the AUC estimates had 95% confidence intervals that included 0.5, indicating that discrimination beyond chance could not be demonstrated in this cohort; together with low sensitivity, these findings limit the utility of EB as a standalone diagnostic tool for these conditions. Although the AUC for AEH+ (atypical endometrial hyperplasia and above) was comparatively higher, its wide confidence interval and the low sensitivity observed for detecting AEH+ support cautious clinical interpretation. In addition, ROC curves exhibited diagonal segments, consistent with overlapping test results between outcome-positive and outcome-negative groups and reflecting limited separability. Collectively, these results suggest that pre-operative EB has a restricted role in predicting final hysterectomy pathology when used in isolation, and should be interpreted in conjunction with imaging findings, clinical characteristics, and complementary diagnostic methods during clinical decision-making.

Another noteworthy finding of our study concerns PMK cases. None of the 83 patients who underwent EB due to PMB and whose results were reported as benign or inadequate showed malignancy in the final pathology after hysterectomy. This situation supports the notion that the negative predictive value of EB in ruling out malignancy is quite high in post-menopausal patients and that a benign biopsy result in these cases provides reliable information to the clinician.

In light of these findings, a benign biopsy result may not be sufficient on its own to rule out malignancy, particularly in symptomatic patients with risk factors (obesity, advanced age, diabetes mellitus, unopposed oestrogen exposure, etc.). Therefore, even if the histopathological result is benign, in cases where AUB persists or clinical suspicion is strong, it would be a safer clinical approach not to terminate the diagnostic process and to include hysteroscopy or advanced imaging methods that allow direct evaluation of the uterine cavity.

### 4.7 Limitations of endometrial biopsy in the diagnosis of focal lesions

The limited sensitivity of EB is also observed in the diagnosis of focal intrauterine lesions such as endometrial polyps and chronic endometritis. All blind endometrial sampling methods are generally unreliable in the diagnosis of endometrial polyps and have been shown to miss most focal lesions (12, 53). It has been reported that sensitivity is below 30% for endometrial polyps in particular and that there is inconsistency with the final histopathology (54, 55).

In our study, EB was found to have a sensitivity of 30.6% and a specificity of 83% in the pre-operative diagnosis of polyps. These rates indicate that EB has a very limited role in the diagnosis of endometrial polyps. These low rates can be explained by the fact that the lesions cannot be detected by blind techniques due to their focal distribution. Consequently, cases of endometrial carcinoma developing on the basis of endometrial polyps may be missed. Unlike blind techniques, hysteroscopy is currently considered the gold standard for detecting focal intrauterine pathology and obtaining a reliable histological diagnosis of endometrial polyps due to its ability to directly visualise the lesion (12, 53, 56).

In our study, the reliability of EB in the diagnosis of chronic endometritis was found to be similar to that of endometrial polyps. Although its specificity was 94.7%, higher than that of endometrial polyps, its sensitivity of 30% alone was found to be insufficient for the diagnosis of endometritis.

### 4.8 The clinical significance of false negative results in endometrial biopsy

The finding that patients diagnosed with functional endometrium by EB ultimately harbour abnormalities such as polyps, hyperplasia, endometritis and even endometrial carcinoma in the final pathology results highlights the significant clinical consequences of false negative EB results (57).

In our study, endometrial carcinoma was detected in the final pathology in two cases that were similarly reported as benign by EB. Furthermore, in three cases, the pre-operative diagnosis was upgraded to AEH+, and in nine cases, it was upgraded to Non-AEH. These results confirm that blind endometrial sampling methods may miss concomitant malignancies, particularly in focal lesions (20, 57). Particular care should be taken in the management of patients who are resistant to medical treatment, carry risk factors, and have EB results that are inconsistent with clinical findings (58). If clinical suspicion persists despite negative EB results, it is critically important to perform advanced investigations such as transvaginal ultrasonography (TV-USG), saline infusion sonohysterography (SIS), hysteroscopy, CT, and MRI (59). Consequently, it would be more appropriate to use EB as an exclusion test rather than as a definitive diagnostic tool (60).

## 5 Strengths of the study

The most significant strength of our study is that all pre-operative biopsy data have been validated by definitive hysterectomy pathologies, which are considered the gold standard for diagnosis, thereby eliminating verification bias. Additionally, standardising the aetiological distribution based on the current FIGO PALM-COEIN system and comprehensively examining post-menopausal cases as a separate clinical entity enhances the scientific value of our study and its contribution to the literature.

## 6 Limitations

Our study has several limitations inherent to its design. First, the retrospective nature and single-center setting may limit the generalizability of our findings. Second, due to the structure of the hospital electronic records, we could not systematically distinguish between patients who underwent Pipelle biopsy versus D&C; therefore, a subgroup analysis comparing their diagnostic performance could not be performed. Third, data on potential confounders such as Body Mass Index (BMI) and comorbidities were not consistently available. Additionally, the lack of data on inter-observer reliability among pathologists represents another constraint. Finally, since our cohort included only patients who underwent hysterectomy, the results may not fully reflect the diagnostic performance of endometrial biopsy in patients managed conservatively.

## 7 Conclusion

Our study confirms the pre-dominant role of leiomyomas in the aetiology of AUB and demonstrates the high specificity of pre-operative EB in ruling out pathologies. Particularly in cases with PMK, benign EB results were found to have a high negative predictive value in ruling out malignancy. However, the low sensitivity observed in focal lesions such as AEH+, endometritis, and endometrial polyps increases the risk of false negatives due to blind sampling methods. Therefore, EB should be considered a powerful screening test rather than a definitive diagnostic tool. In clinical practice, in the presence of risk factors, persistent symptoms, or EB results inconsistent with clinical findings, the diagnostic approach should not be limited to EB despite benign histopathology; further diagnostic evaluation of the uterine cavity, cervix, and adnexa should be performed. In the future, it will be important to validate these findings in different AUB subgroups through multicentre, prospective studies.

## Data Availability

The raw data supporting the conclusions of this article will be made available by the authors, without undue reservation.
